# Characterization and Secretory Expression of a Thermostable Tannase from Aureobasidium melanogenum T9: Potential Candidate for Food and Agricultural Industries

**DOI:** 10.3389/fbioe.2021.769816

**Published:** 2022-02-08

**Authors:** Lu Liu, Jing Guo, Xue-Feng Zhou, Ze Li, Hai-Xiang Zhou, Wei-Qing Song

**Affiliations:** ^1^ Department of Clinical Laboratory, Qingdao Municipal Hospital, Qingdao, China; ^2^ School of Medicine and Pharmacy, Ocean University of China, Qingdao, China; ^3^ Clinical Trial Research Center, The Affiliated Central Hospital of Qingdao University, Qingdao, China; ^4^ College of Advanced Agricultural Sciences, Linyi Vocational University of Science and Technology, Linyi, China

**Keywords:** tannin, tannase, thermostability, *Aureobasidium melanogenum*, *Yarrowia lipolytica*

## Abstract

Being a key industrial enzyme, tannase is extensively applied in various fields. Despite the characterizations of a large number of tannases, there are hardly a few tannases with exceptional thermostability. In this detailed study, a tannase-encoding gene named *tanA* was identified from *Aureobasidium melanogenum* T9 and heterologously expressed in *Yarrowia lipolytica* host of food grade. The purified tannase TanA with a molecular weight of above 63.0 kDa displayed a specific activity of 941.4 U/mg. Moreover, TanA showed optimum activity at 60°C and pH 6.0. Interestingly, TanA exhibited up to 61.3% activity after incubation for 12 h at 55°C, signifying its thermophilic property and distinguished thermostability. Additionally, TanA was a multifunctional tannase with high specific activities to catalyze the degradation of various gallic acid esters. Therefore, this study presents a novel tannase, TanA, with remarkable properties, posing as a potential candidate for food and agricultural processing.

## Introduction

As a type of polyphenol compound generated to resist a bad growth environment, tannins are extensively present in higher plants (Chung et al., 1998). The formation of stable complexes with various biological macromolecules, such as polysaccharides and proteins, provides lower nutritive value to food, thus making them nutritionally undesirable (Pan et al., 2020). According to some reports, tannins are one of the primary causes of low food intake, slow growth, low level of fodder utilization rate, and low protein breakdown in laboratory animals (Becker and Makkar, 1999; Min et al., 2005; Sengil and Oezacar, 2009). Therefore, the removal of tannins becomes an integral process during food and feed processing. Fortunately, there still exist some special microorganisms which can grow and reproduce stably in an environment containing tannins, making the full utmost of the secreted enzymes to decompose tannins into small-molecule phenolic compounds (Chhokar et al., 2013).

Tannase (EC 3.1.1.20), also known as tannin acylhydrolase, is a hydrolase that can effectively hydrolyze ester bonds and carboxyphenol bonds in hydrolyzable tannins, including ellagitannins (ETs), gallotannins (GTs), and other gallic acid esters to produce polyphenol compounds such as gallic acid (Aguilar and Gutierrez-Sanchez, 2001; Zhang et al., 2015; Govindarajan et al., 2016). The enzymatic degradation of tannins is considered a safe and environmentally friendly method that can effectively overcome the shortcomings of traditional hydrolysis methods and significantly improve the yield and purity of products (Raghuwanshi et al., 2011; Jana et al., 2013; Ghosh and Mandal, 2015; Ebrahimzade et al., 2018). Therefore, tannases have played a significant role in industrial production, especially in the fields of feed, food, brewing, and pharmaceuticals (Chhokar et al., 2013; Wang et al., 2022). Rapeseed meal is considered to be one of the sources of high-quality plant protein feed due to its high protein content and balanced amino acid composition. However, rapeseed meal contains a variety of anti-nutritional factors, like tannins, that seriously affect its palatability as well as nutritive value, severely limiting the application of rapeseed meal in animal feeding (Shim et al., 2003). Interestingly, Lorusso *et al*. (1996) reported that the tannase produced by *Trametes versicolor* could degrade more than 80% of tannins in rapeseed meal within 30 min, strengthening the application of tannase in the processing of rapeseed meal feed. Furthermore, the transformation of ester catechins (also belonging to tannins) in green tea to non-ester catechins for improving the taste of tea drinks is another important use for tannases (Ozturk et al., 2016). Therefore, these properties of tannases are of great interest among researchers.

Currently, owing to the characteristics of biochemical diversity and liable genetic manipulations, microbial fermentation is the major approach for the continuous production of tannases (Beniwal et al., 2013). Numerous studies have been conducted for screening tannase-producing microorganisms, of which bacteria and fungi have shown enhanced enzyme production ability (Banerjee et al., 2012; Kanpiengjai et al., 2019). However, tannases produced by bacteria demonstrated limitations in low activity and poor thermostability, imposing major concerns of large-scale application (Rivas et al., 2019). Thus, there are relatively scarce studies of bacterial tannases. On the other hand, the complex heredity of fungi makes genetic manipulations difficult (Beniwal et al., 2010). These collectively present challenges in identifying tannases with better properties for industrial application—for example, tannases play a pivotal role in green tea deep processing to improve the appearance, aroma, and flavor of tea and the extraction efficiency of polyphenols (Cao et al., 2019). During the extraction of green tea, the extraction yield increased with increasing temperature within a certain range (Shao et al., 2020). However, since the enzyme catalysis reaction is usually temperature-limited, tea processing and extraction consume a prolonged time (over 2 h) at a lower range of temperature from 30 to 40°C, thereby reducing product quality and production efficiency (Hong et al., 2013). Therefore, it becomes necessary to screen genetically stable microorganisms with the capability to produce tannases of high vigor and thermal stability. Jana *et al*. (2013) reported that the high-temperature and solvent-resistant tannase produced from *Bacillus subtilis* PAB2 had a half-life of up to 4.5 h in an environment of 60°C. Shao *et al*. (2020) screened *Aspergillus niger* FJ0118 that could generate tannase rAntan1 with strong temperature tolerance, and its half-life at 60°C was persisting for about 5.4 h. Nevertheless, the majority of the reported thermostable tannases cannot reach food grade due to safety concerns.

*Aureobasidium melanogenum* T9, with the capability to degrade tannins, has been successfully isolated from the starter of red wine (Zhang et al., 2019). In this study, the gene of encoding tannin-degrading enzyme, *tanA*, was identified and heterologously expressed in the *Yarrowia lipolytica* yeast of food grade. The detailed characterization of tannase TanA displayed superior thermal stability and unique robustness, thus laying a solid foundation for its application in the industry.

## Materials and Methods

### Bioinformatics Analysis of TanA

In the previous research, the genome of *A. melanogenum* T9 strain was sequenced and annotated by Novogene (Zhang et al., 2019); *in silico* studies were performed to determine the encoding gene sequence of TanA. By using HMMER3, the protein sequence was compared with carbohydrate-active enzyme database to acquire the message. The filter condition was set to *E* value <1 E^−5^. The theoretical molecular weight (Mw) and isoelectric point (pI) value were obtained through online prediction (http://web.expasy.org/compute_pi/). SignalP 4.1 server was applied for signal peptide analysis (http://www.cbs.dtu.dk/services/SignalP-4.1/). NetNGlyc 1.0 server was applied to predict N-glycosylation sites (http://www.cbs.dtu.dk/services/NetNGlyc/). The conserved domain database of the National Center for Biotechnology Information (NCBI, Bethesda, MD, United States) was used for domain analysis (https://www.ncbi.nlm.nih.gov/cdd).

To study the evolutionary relationship among TanA and other tannases derived from microorganisms, a bootstrapped phylogenetic tree was constructed by neighbor-joining method with MEGA 6.0 software (Tamura et al., 2013) on the basis of the amino acid sequences of related tannases obtained from NCBI (https://www.ncbi.nlm.nih.gov/). Multiple sequence alignment was carried out using DNAMAN 6.0 software (Lynnon Biosoft, Foster City, CA, United States). The 3D model of tannase TanA (accession no.: QEP28943.1) of *A. melanogenum* T9 was built on the basis of the crystal structure of feruloyl esterase AoFaeB from *Aspergillus oryzae* RIB40 (PDB: 3WMT) (Suzuki et al., 2014), using basic modeling module in modeller 9.20. Fifty homologous models were firstly constructed, and one model with the highest score was obtained.

### Secretory Expression of TanA

The *tanA* gene without signal sequence was synthesized by Synbio Technologies (Suzhou, China) following codon optimization. The linearized DNA fragment was successfully transformed into the *Y. lipolytica* URA^−^ strain (Zhang et al., 2018). The *Y. lipolytica* cultivation and the purification steps of TanA generally followed those of Zhou *et al*. (2020). After culturing in GPPB liquid media [30.0 g/L glucose, 2.0 g/L yeast extract, 1.0 g/L (NH_4_)_2_SO_4_, 3.0 g/L K_2_HPO_4_, 2.0 g/L KH_2_PO_4_, 0.1 g/L MgSO_4_·7H_2_O, pH 6.8] for 72 h at 30°C, the tannase activities of the positive transformants were discovered (Pan et al., 2020). In comparison, the recombinant strain 72 displayed optimal extracellular activity. Afterwards, 100 ml of the supernatant was concentrated firstly by ultrafiltration with a centrifugal filter 3 K device (Millipore, Burlington, MA, United States), and then it was injected into the His60 Ni Superflow affinity chromatography column purchased from Clontech Laboratories, Inc. (TaKaRa, Dalian, China), which was already equilibrated with 50 mM phosphate buffer (pH 7.4) containing 300 mM NaCl and 20 mM imidazole. After successive washing using the same buffer containing 40 mM imidazole and elution using the same buffer containing a linear gradient of imidazole (50–400 mM), the 6×His-tagged TanA was obtained (Zhang et al., 2021). The fractions with tannase activity were pooled and concentrated *via* Millipore centrifugal filter 3 K device. Meanwhile, the enzyme solution was desalted, and the buffer was replaced with 50 mM Tris-HCl (pH 7.4) for further characterization of TanA. During the purification of TanA, a BCA protein assay kit (TaKaRa, Dalian, China) was used to determine the total protein concentration. Moreover, 12% sodium dodecyl sulfate–polyacrylamide gel electrophoresis (SDS-PAGE) was used to verify the Mw and purity of TanA.

### Determination of Tannase Activity

TanA activity was determined by measuring the amount of gallic acid produced in the reaction, generally according to the description in Sharma *et al*. (2000). Briefly, 0.5 ml of properly diluted enzyme solution was mixed with 4.5 ml of 0.5% (w/v) propyl gallate in 100 mM Na_2_HPO_4_–citric acid buffer (pH 5.0) at 40°C for 10 min. The gallic acid content was measured by the formation of chromogen violet staining between 0.667% (w/v) rhodanine in ethanol and gallic acid, followed by recording the absorbance at 520 nm by a spectrophotometer. One unit of enzyme activity was defined as the amount of tannase that generates 1 μmol gallic acid per minute.

### Temperature and pH Properties for TanA Activity

The optimum temperature was determined by carrying out a TanA-mediated hydrolysis reaction at different temperatures ranging from 20 to 70°C. For the purpose of assessing thermostability, the residual activity of purified TanA was investigated after incubating at 20–70°C for 12 h. Additionally, propyl gallate solutions were prepared with 100 mM disparate pH buffers (glycine–NaOH, pH 8.5–11.0; Na_2_HPO_4_–citric acid, pH 2.0–8.0) to conduct as substrates of enzymatic reaction for the confirmation of optimum pH. The pH stability of TanA was surveyed through measuring the rest of enzyme activity after incubation at 40°C for 12 h in buffers of different pH.

### Effects of Chemicals and Metal Ions on TanA Activity

The catalytic reactions were performed using propyl gallate liquors with 1 and 5 mM of different compounds or metal ions as substrates. The reaction that took place in a propyl gallate solution without any compound/metal ion was considered the control.

### Degradation of Gallic Acid Esters by TanA

The substrate specificity of tannase TanA was analyzed according to its specific activities assayed under the standard method described above using 0.5% (w/v) of different gallic acid esters as substrates, including methyl gallate (MG), propyl gallate (PG), tannic acid (TA), gallocatechin gallate (GCG), epicatechin gallate (ECG), and epigallocatechin gallate (EGCG). The chemical structures of the substrates are displayed in Supplementary Figure S1. Meanwhile, the Michaelis constants (*K*_m_) were calculated against these substrates based on the double-reciprocal plot of Lineweaver–Burk (Lineweaver and Burk, 1934).

In order to further investigate the capability of TanA to degrade these substrates, excess tannase TanA (0.5 U per milligram of each substrate, namely, 2.5 U/ml of each reaction solution) was incubated with 0.5% (w/v) of each gallic acid ester (pH 6.0); the catalytic reaction mixtures were stirred continuously at 55°C for 2 h. After incubation, the solution of each degraded product was immediately loaded onto the Millipore centrifugal filter 3 K device to remove the protein and unsolved materials. High-performance liquid chromatography (HPLC) was employed to confirm the transformation of these substrates, and the analysis method was described briefly as follows: Diamonsil C_18_ column (3.0 × 250 mm, 5 μm), wavelength of 278 nm, mobile phase A (acetonitrile) and B (0.5% acetic acid–water), and flow velocity of 1.0 ml/min by gradient elution as reported (Ni et al., 2015).

## Results

### Bioinformatics Analysis of TanA

**FIGURE 1:**
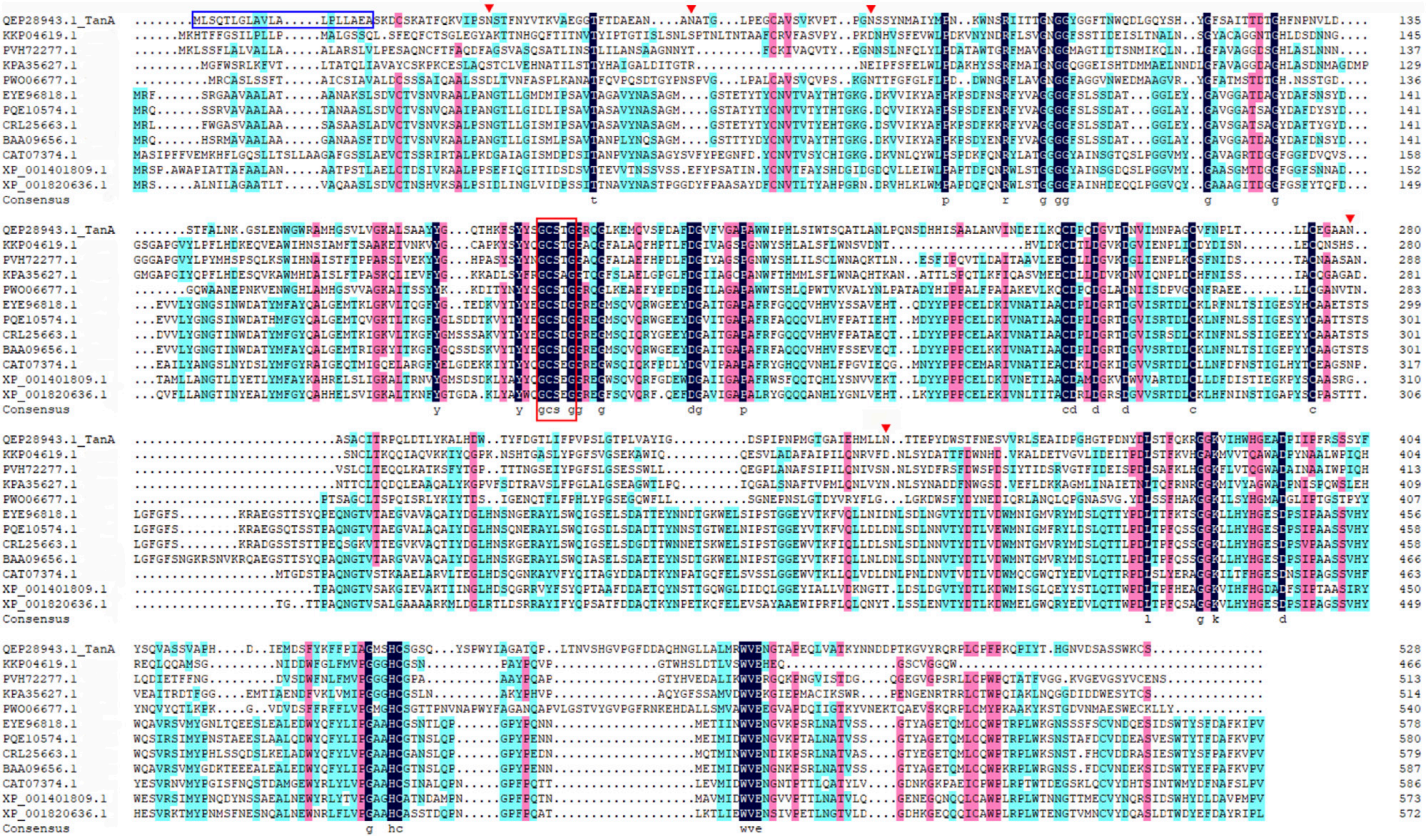
Multiple sequence alignment among TanA and other reported tannases. The signal peptide is boxed in blue, the Gly-X-Ser-X-Gly conserved domain is highlighted by a red box, and the predicted N-glycosylation sites are marked with red inverted triangles.

The genomic DNA of *A. melanogenum* T9 strain sequencing illustrated a putative tannase-encoding gene *tanA*. The deduced amino acid sequence was deposited into the Genebank database, and the accession number QEP28943.1 was provided (Zhang et al., 2019). The open reading frame of *tanA* was composed of 1,587 bp encoding a protein of 528 amino acids, of which the first 19 amino acids (highlighted by the blue box in Figure 1) were anticipated as the signal peptide, which was consistent with the secretion feature. The theoretical pI and Mw were predicted to be 5.50 and 57.2 kDa, respectively. Interestingly, the BLAST tool of NCBI revealed that TanA possessed the conserved domain closely related to the tannase and ferulic acid esterase family (data not shown).

**FIGURE 2:**
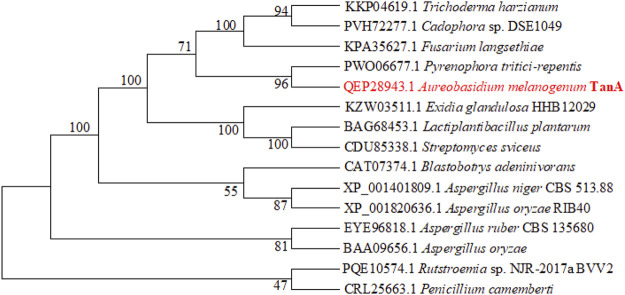
The phylogenetic tree constructed according to the sequences of TanA and other tannases. TanA researched in this study is in red.

Then, a phylogenetic tree was built to determine the ascription of TanA. As illustrated in Figure 2, the present enzyme TanA, which was on the same branch as the tannase from *Pyrenophora tritici-repentis* (accession no.: PWO06677.1), distinctly pertained to the tannase and feruloyl esterase family. Moreover, sequence comparative analysis was performed among TanA and other members of the tannase and feruloyl esterase family (Figure 1), including the tannase from *Trichoderma harzianum* (accession no.: KKP04619.1), the tannase from *Cadophora* sp. DSE1049 (accession no.: PVH72277.1), the tannase from *Fusarium langsethiae* (accession no.: KPA35627.1), the tannase from *P. tritici-repentis* (accession no.: PWO06677.1), the tannase from *Aspergillus ruber* CBS 135680 (accession no.: EYE96818.1), the tannase from *Rutstroemia* sp. NJR-2017a BVV2 (accession no.: PQE10574.1), the tannase from *Penicillium camemberti* (accession no.: CRL25663.1), the tannase from *A. oryzae* (accession no.: BAA09656.1), the tannase from *Blastobotrys adeninivorans* (accession no.: CAT07374.1), the tannase from *A. niger* CBS 513.88 (accession no.: XP_001401809.1), and the tannase from *A. oryzae* RIB40 (accession no.: XP_001820636.1). The multiple sequence alignment showed that TanA retained a typical Gly-X-Ser-X-Gly conserved domain (represented by the red box in Figure 1). The 3D structure of TanA in this study has been constructed through homology modeling, and the molecular graphic image was prepared using PyMOL 2.0.3 (Schrödinger, LLC, Portland, OR, United States) and displayed in Supplementary Figure S2.

### Secretory Expression of TanA in *Y. lipolytica*

**FIGURE 3:**
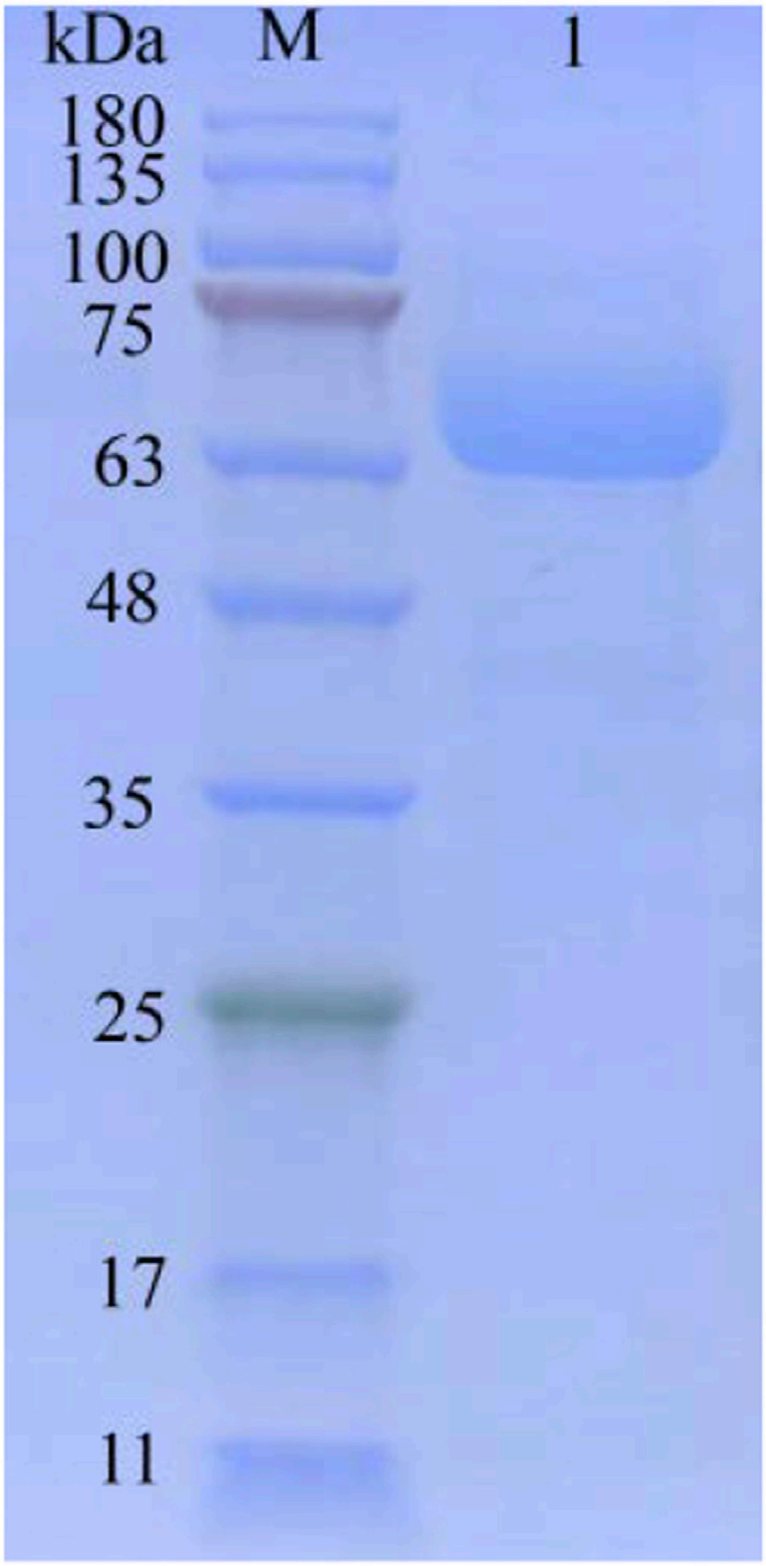
SDS-PAGE of purified TanA. Lane 1: purified TanA; lane M: prestained protein ladder.

The secretory expression of TanA was further studied in the heterogenous host of food grade with significant cellular exocrine ability, *Y. lipolytica* (Madzak, 2015). The tannase activity was measured as 89.4 U/ml following 72 h of incubation in GPPB medium. The summary of TanA purification is listed in Table 1. After Ni-IDA affinity chromatography, the purified TanA was obtained, with a specific activity of 941.4 U/mg. The SDS-PAGE analysis of the purified TanA protein depicted a predominant band observed at approximately 63.0 kDa (Figure 3), a bit larger than the theoretical calculation, which could be possibly due to the glycosylation of the protein. To this end, NetNGlyc 1.0 server was employed to forecast five N-glycosylation recognition sites of TanA (marked with red and inverted triangles in Figure 1). In addition, the fusion of 6×His-tag introduced several extra amino acids into the recombinant TanA.

**TABLE1:** Purification of tannase TanA.

Purification step	Total protein (mg)	Total activity (U)	Specific activity (U/mg)	Purification fold	Yield (%)
Crude enzyme	57.5	8,940.0	155.5	1	100
Ni-IDA agarose	5.81	5,469.5	941.4	6.05	61.2

### Effects of Temperature on TanA Activity

**FIGURE 4:**
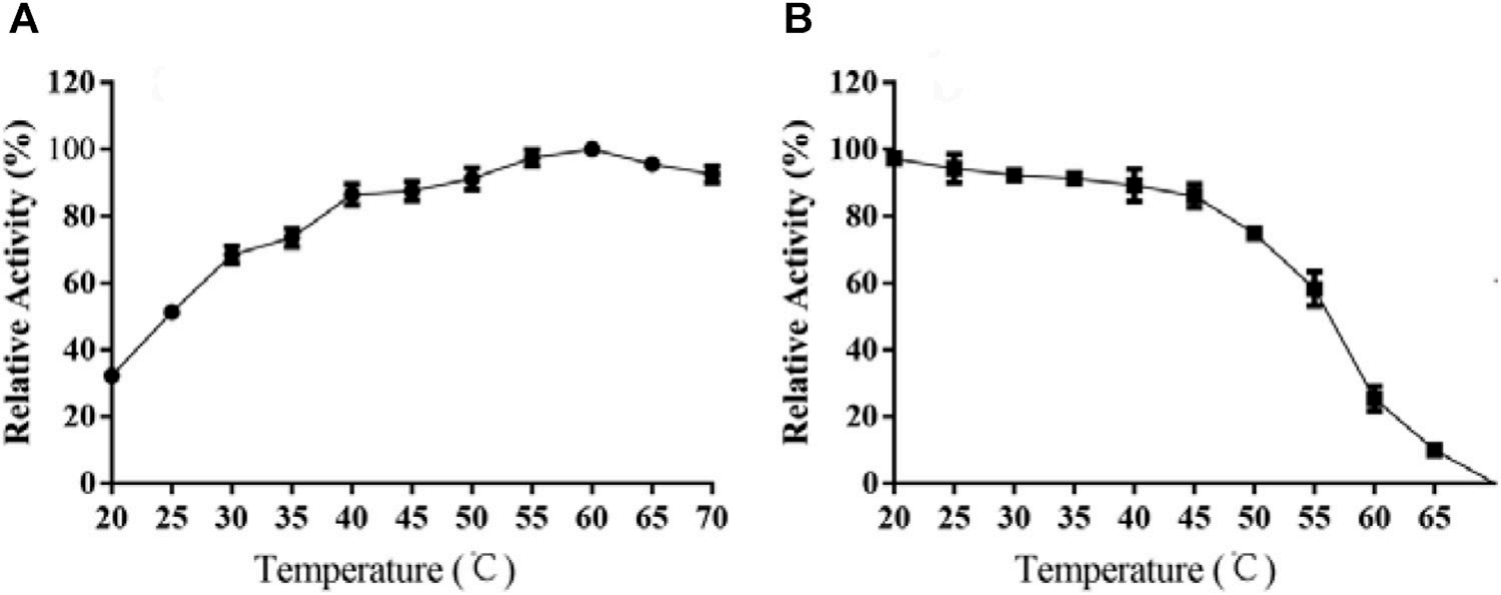
Effects of temperature on the activity **(A)** and stability **(B)** of TanA. **(A)** The optimal temperature of TanA was assessed in the range of 20–70°C, regarding the activity at optimum temperature as 100%. **(B)** The temperature stability of TanA was determined by measuring the residual activity after incubation under 20–70°C for 12 h; the initial activity was taken as 100%.

As demonstrated in Figure 4A, the activity of TanA was linearly correlated to temperature rise, with optimal response at 60°C and over 80% activity retention from 40 to 70°C. Interestingly, TanA still showed 85.2 and 81.5% of optimum activity even under the conditions of 65 and 70°C (Figure 4A). It was further observed that TanA exhibited impressive stability below 55°C. It could maintain up to 61.3% activity even after 12 h of incubation at 55°C (Figure 4B). Therefore, TanA demonstrated thermophilic property with superior thermostability.

### Effects of pH on TanA Activity

**FIGURE 5:**
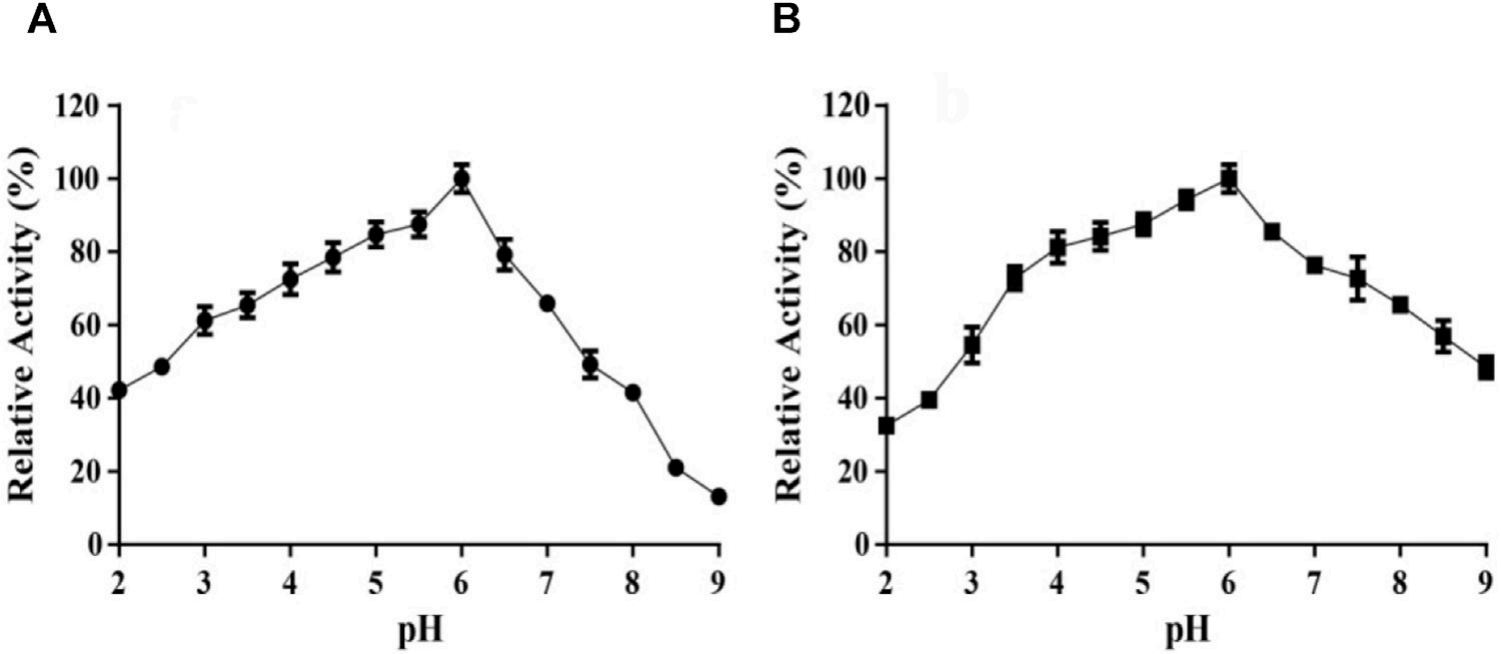
Effects of pH on the activity **(A)** and stability **(B)** of TanA. **(A)** The optimal pH of TanA was investigated in the pH range of 2–11 with 100 mM buffers (glycine–NaOH, pH 8.5–11.0; Na_2_HPO_4_–citric acid, pH 2.0–8.0) by taking the activity at the optimum pH as 100%. **(B)** The pH stability of TanA was surveyed after incubation for 12 h at 40°C in the pH range of 2–11 with the buffers described above; the highest residual activity was set as 100%. The indifferent data ranging from pH 9 to 11 were neglected and not shown in both figures **(A)** and **(B)**.

As TanA displayed the optimal catalytic activity at pH 6.0 (Figure 5A), the effects of pH was elucidated. TanA maintained exceeding 70% of its activity after incubation for 12 h at 40°C within the wide pH range from 3.5 to 7.5 (Figure 5B), indicating that it possessed favorable stability under acidic to weakly alkaline conditions.

### Effects of Chemicals or Metal Ions on TanA Activity

**FIGURE 6:**
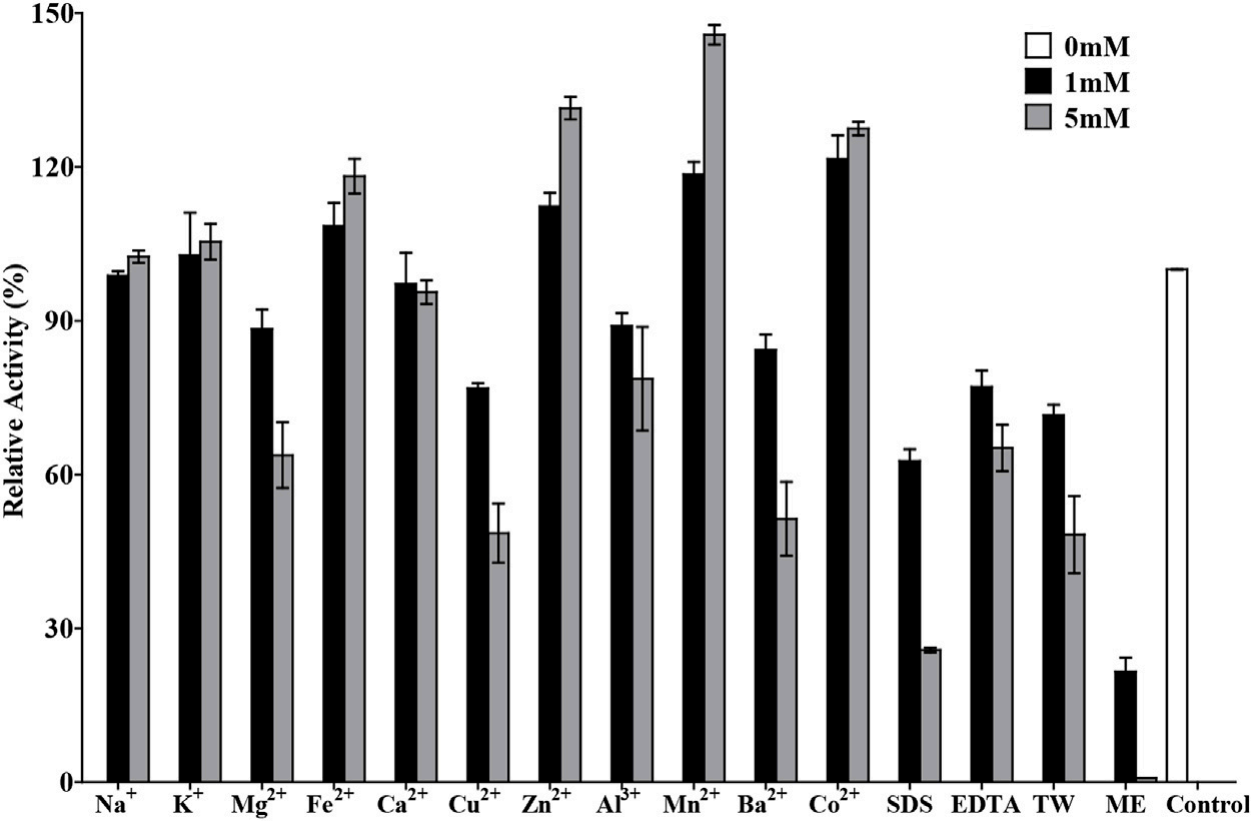
Effects of chemicals and metal ions on the activity of TanA. The activity assayed without any chemical compound or metal ion was taken as control. SDS, sodium dodecyl sulfate; EDTA, ethylene diamine tetraacetic acid; TW, Tween 80; ME, mercaptoethanol.

The effects of various metal ions and chemicals on the activity of TanA are shown in Figure 6. Cu^2+^, Ba^2+^, Al^3+^, and Mg^2+^ strongly inhibited the activity of TanA. However, a slightly activated effect was observed in the presence of Fe^2+^; the relative activity reached 118.2% with 5 mM Fe^2+^. Contrastingly, the enzymatic activity was obviously enhanced by Zn^2+^, Mn^2+^, and Co^2+^ at both 1 and 5 mM, which was increased to 131.5, 145.8, and 127.5%, respectively, with 5 mM of these cation ions. Other chemical compounds, especially mercaptoethanol (ME), manifested dramatic inactivation effects on TanA, whose activity was reduced to 21.6% compared to the control when subjected to 1 mM of ME and even was thoroughly lost after 5 mM of ME was added into the reaction solution, which perhaps is attributed to the significant ability of ME to damage disulfide bonds.

### Substrate Specificity of TanA and Degradation Product Analysis

In order to study the substrate specificity of TanA, various gallic acid esters were employed as substrates. The specific activities and *K*_m_ values of TanA against these substrates are listed in Table 2. TanA showed multifunctional property with high specific activities and affinities toward all the gallic acid esters utilized. Although the synthetic substrate PG demonstrated the highest specific activity of TanA at 941.4 U/mg, it still exhibited considerable catalysis ability against other substrates, especially toward some natural substrates, such as GCG, ECG, and EGCG (Table 2). For further testing of the abilities of TanA to degrade diverse gallic acid esters, the HPLC analysis of degradation products is displayed in Supplementary Figure S3 . After catalytic decomposition by TanA at 55°C for 2 h, no gallic acid ester could be detected in the reaction solutions, and all kinds of substrates were almost thoroughly transformed (Supplementary Figure S3). In the chromatograms of the transformation products after tannase TanA treatment against MG (Supplementary Figure S3A), PG (Supplementary Figure S3B), and TA (Supplementary Figure S3C), only the peaks of gallic acid could be revealed due to other degraded products (methanol for MG, propanol for PG, and glucose for TA) with no ultraviolet absorption. Significantly, the ester catechins that are ubiquitous in green tea, including GCG (Supplementary Figure S3D), ECG (Supplementary Figure S3E), and EGCG (Supplementary Figure S3F), were converted successfully to gallocatechin (GC), epicatechin (EC), and epigallocatechin (EGC), respectively, which belong to the non-eater catechins.

**TABLE 2:** Comparison of substrate specificities for TanA.

Substrate	Specific activity (U/mg)	*K*_m_ (mM)
TA	723.8	2.15
GCG	703.1	1.86
ECG	756.2	2.13
PG	941.4	1.71
MG	506.8	2.81
EGCG	623.2	2.52

TA, tannic acid; GCG, gallocatechin gallate; ECG, epicatechin gallate; PG, propyl gallate; MG, methyl gallate; EGCG, epigallocatechin gallate.

## Discussion

The previous study of the genes related to tannin degradation in *A. melanogenum* T9 has suggested that there existed several tannases (including TanA, TanB, and TanC) with different molecular masses, amino acid sequences, gene expression patterns, and diverse properties, which are together responsible for the catalyzed hydrolysis of tannins in the strain T9 (Zhang et al., 2019). The tannases from different categories are often produced in the same fungal species due to species-specific accumulative effects and the impacts of exterior circumstances. Tannase subtypes are distinguished depending on their physical and chemical properties, such as molecular size, family, presence or absence of a signal peptide, and more, which guarantee that microorganisms are able to degrade both intra- and extracellular tannins under various physiological conditions, namely, temperature, pH, ion strength, *etc*. Therefore, it is of great significance to figure out the metabolic mechanism of tannins in *A. melanogenum* in future work.

TanA exhibited a typical conserved domain, Gly-X-Ser-X-Gly, almost present in all tannase sequences of fungi and bacteria (boxed in red in Figure 1). This unique sequence of serine hydrolase effectively binds to the tannic substrates, thereby promoting catalytic activity (Zhang et al., 2019). Moreover, a mutation analysis explained that the disulfide bond between Cys202 and Cys458 played a vital part in regulating the activity of AoFaeB (Suzuki et al., 2014). Interestingly, TanA also showed several cysteine residues probably involved in disulfide bond formation at similar sites as that of AoFaeB. For the sake of surveying this, the homology model of TanA was built. Meanwhile, four disulfide bonds with a 3D structure have been predicted (Supplementary Figure S2), including Cys161–Cys415, Cys230–Cys247, Cys256–Cys265, and Cys487–Cys508, among which the first disulfide bond was similar to the one in AoFaeB as described above, indicating that the disruption of disulfide bonds when subjected to ME affected TanA activity and stability to a great extent (Figure 6).

It is shown in Figure 4A that TanA displayed an optimal response at 60°C, with over 80% relative activity from 40 to 70°C, illustrating its thermophilic property and making it suitable for most of the hydrolysis processes assisted by tannases which were carried out at increased temperatures (Abdulhameed et al., 2005). However, a lot of studies demonstrated that many tannases from bacteria (Jana et al., 2014), mold (Iibuchi et al., 1968; Lekha and Lonsane, 1997; Madzak, 2015; Sivashanmugam and Jayaraman, 2011), and even yeast (Wang et al., 2020) showed optimal activity at approximately 40°C. Considering thermal stability, as exhibited in the diagram of Figure 4B, TanA depicted superior heat resistance property at a temperature below 55°C, maintaining up to 61.3% activity even after 12 h of incubation. Studies showed thermostability in the range of 30–50°C for most fungal tannases (Table 3). TanA possessed better temperature stability compared to other tannases from fungi. Although the tannase from *P. notatum* NCIM923 (Gayen and Ghosh, 2013) and the tannase from *K. marxianus* NRRL Y-8281 (Mahmoud et al., 2018) could maintain their stability up to 60 and 70°C, respectively, according to their researches (Table 3), their thermostability at high temperatures was, in fact, worse than TanA. The purified tannase from *P. notatum* retained 60% of its thermostability at 60°C within only 1 h (Gayen and Ghosh, 2013). Meanwhile, the tannase from *K. marxianus* could also remain stable at 70°C for only 60 min (Mahmoud et al., 2018). Also belonging to thermophilic tannases, the extracellular tannase derived from *A. phoenicis*, whose optimal temperature was the same as TanA, was stable for 1 h at 40–50°C, with a half-life of only 20 min at 60°C (Riul et al., 2013). In industrial production such as green tea deep processing, the degradation of tannins often takes a longer time at a relatively high temperature, and the tannase TanA retained most of its activity within at least 12 h under 55°C. This concludes that TanA is a thermophilic tannase with sturdy thermostability, which quite better satisfy the industrial application. Contrastingly, the optimal temperature for the tannase rAntan1, with strong thermal stability from *A. niger* FJ0118, was 80°C. It could persist to be stable at 60°C for about 5.4 h (Shao et al., 2020). Although owning favorable activity and stability at high temperature, rAntan1 expressed by *Pichia pastoris* cannot reach food grade because of the use of methanol when inducing tannase expression. The thermophilic and thermostable properties of TanA and rAntan1 may be partially attributed to glycosylated formation during expression in yeast (Zhou et al., 2020), corresponding to the predicted N-glycosylation recognition sites (Figure 1). Yang *et al*. (2007) indicated that the tea steeped in water at 70°C contained the highest contents of active components. Thus, the thermophilic and thermostable tannases, such as TanA and rAntan1, are rare in nature but necessary in industrial production. Some pieces of literature have reviewed the wide application of thermostable tannases in tea extracts to decrease the formation of tea cream (on account of the presence of tannins), thereby improving the taste of tea beverages (Chávez-González et al., 2012). In addition, thermo-tolerant tannases were also applied in the production of instant green tea powder to enhance the color and taste (Ozturk et al., 2016; Cao et al., 2019). In the process of catalytic degradation of tannins, thermostable tannases can enhance high-temperature extraction efficiency, simplify the production process, reduce the costs, and improve the product quality, which are reasons for the application of TanA in industrial production, such as in green tea deep processing.

**TABLE 3:** Biochemical properties of TanA and other reported tannases.

Source	Specific activity (U/mg)	Optimal pH/temperature (°C)	Temperature-stable range (°C)	pH-stable range	References
*Penicillium notatum*	22.48	5.0/40	30–60	3.0–8.0	Gayen and Ghosh (2013)
*Emericella nidulans*	1.91	5.0/45	22–50	4.0–5.0	Goncalves *et al*. (2011)
*Aspergillus phoenicis*	10.0	6.0/60	40–50	2.5–7.0	Riul *et al*. (2013)
*A. niger*	N.D.	6.0/80	30–60	3.0–8.0	Shao *et al*. (2020)
*Sporidiobolus ruineniae*	16.232	7.0/40	20–50	5.0–9.0	Kanpiengjai *et al*. (2020)
*Kluyveromyces marxianus*	1,026.12	4.5, 8.5/35	30–70	4.0–6.0	Mahmoud *et al*. (2018)
*Streptomyces sviceus*	114.0	8.0/50	63–69	6.5–8.0	Wu *et al*. (2015)
*Lactobacillus plantarum*	84.34	8.0/40	N.D.	N.D.	Iwamoto *et al*. (2008)
*A. melanogenum*	941.4	6.0/60	20–55	3.5–7.5	This study

TanA maintained over 60% activity within the broad pH scope of 3.0–7.0, with maximum activity at pH 6.0 (Figure 5A). For pH stability, TanA still retained over 70% of its activity after incubation for 12 h within the wide pH from 3.5 to 7.5, indicating advisable stability under acidic to weakly alkaline conditions (Figure 5B). It was consistent with the reported fungal tannases—for instance, the tannase derived from *A. niger* possessed an optimal pH of 6.0 and excellent stability at pH 3.0–8.0 (Shao et al., 2020). The tannase from *A. oryzae* had an optimum pH of 5.5 along with stability between pH 4.5 and 7.5 and over 80% activity retention (Abdel-Naby et al., 2016). On the other hand, the tannase from *E. nidulans* (pH 4.0–5.0) and the tannase from *Aspergillus thorny* (pH 5.0–6.0) displayed limited pH stability (Goncalves et al., 2011; Tanash et al., 2011). Therefore, remarkable stability at high temperatures and a wide pH range become integral properties of TanA, which reduce the risk of pollution during the fermentation process and thereby establish the foundation for its industrial application in tannin biodegradation and gallic acid preparation.

TanA has been incubated with several gallic acid esters to study the substrate specificity. As shown in Table 2, TanA revealed outstanding specific activities to decompose various substrates, illustrating its multifunctional property. The activity of TanA on synthetic substrate PG was significantly superior, whereas the specific activities on some natural substrates (*e*.*g*., 703.1 U/mg against GCG, 756.2 U/mg against ECG, and 623.2 U/mg against EGCG) were also quite considerable (Table 2). Many reported tannases exhibited quite lower activities toward some ester catechins that naturally exist in green tea, such as ECG and EGCG (Bhoite et al., 2013; Ramírez-Coronel et al., 2003; Sharma et al., 2008). Although Mahmoud *et al*. (2018) stated a high activity toward TA (1,026.12 U/mg), the tannase from *K. marxianus* NRRL Y-8281 was most active on TA (100%), followed by MG (74.3%) and PG (64.5%) and expressing minimum enzymatic activity against EGCG (only 10.5%). Obviously, these enzymes cannot satisfy the application in industrial processing when against natural ester catechins. In addition, the HPLC analysis results of transformation products by TanA further confirmed the almost complete degradation of varying kinds of tannins (especially the natural ester catechins in many plants) at a high temperature within 2 h (Supplementary Figure S3). Previous research reported that immobilized tannase could carry out 98% conversion of TA within 6 h (Mahendran et al., 2006). However, tannase from *Bacillus sphaericus* converted 90.8% TA after as long as 24 h (Raghuwanshi et al., 2011). By contrast, the higher efficiency of TanA, along with high bioconversion, undoubtedly benefited from its favorable activities toward different gallic acid esters. Moreover, plants in nature, such as oilseed rape and many fruits, usually contain various hydrolyzable tannins (GTs and ETs) (Lorusso et al., 1996). Thus, the multifunction property gives the tannase TanA the capability to decompose diverse gallic acid esters, which is fully conducive when against different kinds of tannins in the plants. The prime cause of the bitter taste of tea is the presence of ester catechins (mainly EGCG and ECG) (Shao et al., 2020). The reported tannase rAntan1 displayed similar substrate specificity as TanA. Enzymatic extraction reduced the proportion of easter catechins in tea polyphenols, weakened the bitter taste, and improved the overall taste of the tea beverage (Shao et al., 2020). Similarly, the TanA-mediated extraction processing can also effectively transform the ester catechins to the non-ester ones (such as EGC and EC) in green tea, thereby improving the taste of tea drinks. Besides this, the green tea catechins possess many biological activities, such as antiviral, antibacterial, antitumor, and antioxidant properties, which are closely related to the presence of multiple hydroxyl groups and galloyl groups in their structures (Xu et al., 2021).

Tannins are ubiquitously present in higher plants, and biodegradation by tannases is essential in industrial processing, such as in feed additives (Lorusso et al., 1996), myrobalan juice (Srivastava and Kar, 2009), and green tea (Cao et al., 2019; Shao et al., 2020). In addition, many active substances, such as catechins and gallic acid, employ tannases for their respective extraction (Chhokar et al., 2013). Rapeseed meal processing is a worthy example illustrating the application of tannases. The agro-industrial chain of canola oil production generates a large mass of rapeseed meals but faces challenges during recycling owing to the high content of tannins, resulting in huge wastes (Lorusso et al., 1996; Shim et al., 2003). Fortunately, the efficient decomposition of tannins through enzymatic catalysis aids in the production of rapeseed meal feed with added value (Lorusso et al., 1996). In other words, tannases play significant roles in various fields, such as food, brewing, pharmacy, and feed. However, tannases of food grade with advisable properties are still rather rare but necessary. In this research, a novel thermophilic tannase, TanA, with favorable thermostability was demonstrated, with detailed characterizations suggesting a potent candidate for the food and agricultural industries.

## Data Availability

The datasets presented in this study can be found in online repositories. The names of the repository/repositories and accession number(s) can be found below: https://www.ncbi.nlm.nih.gov/genbank/, QEP28943.1.
