# Supplementary material for: Characterization and Secretory Expression of a Thermostable Tannase from Aureobasidium melanogenum T9: Potential Candidate for Food and Agricultural Industries
Source: Front Bioeng Biotechnol. 2022 Feb 8;9:769816. doi: 10.3389/fbioe.2021.769816 (PMC8861512; doi:10.3389/fbioe.2021.769816)
Supplement: Supplementary file 1 [file DataSheet1.docx]

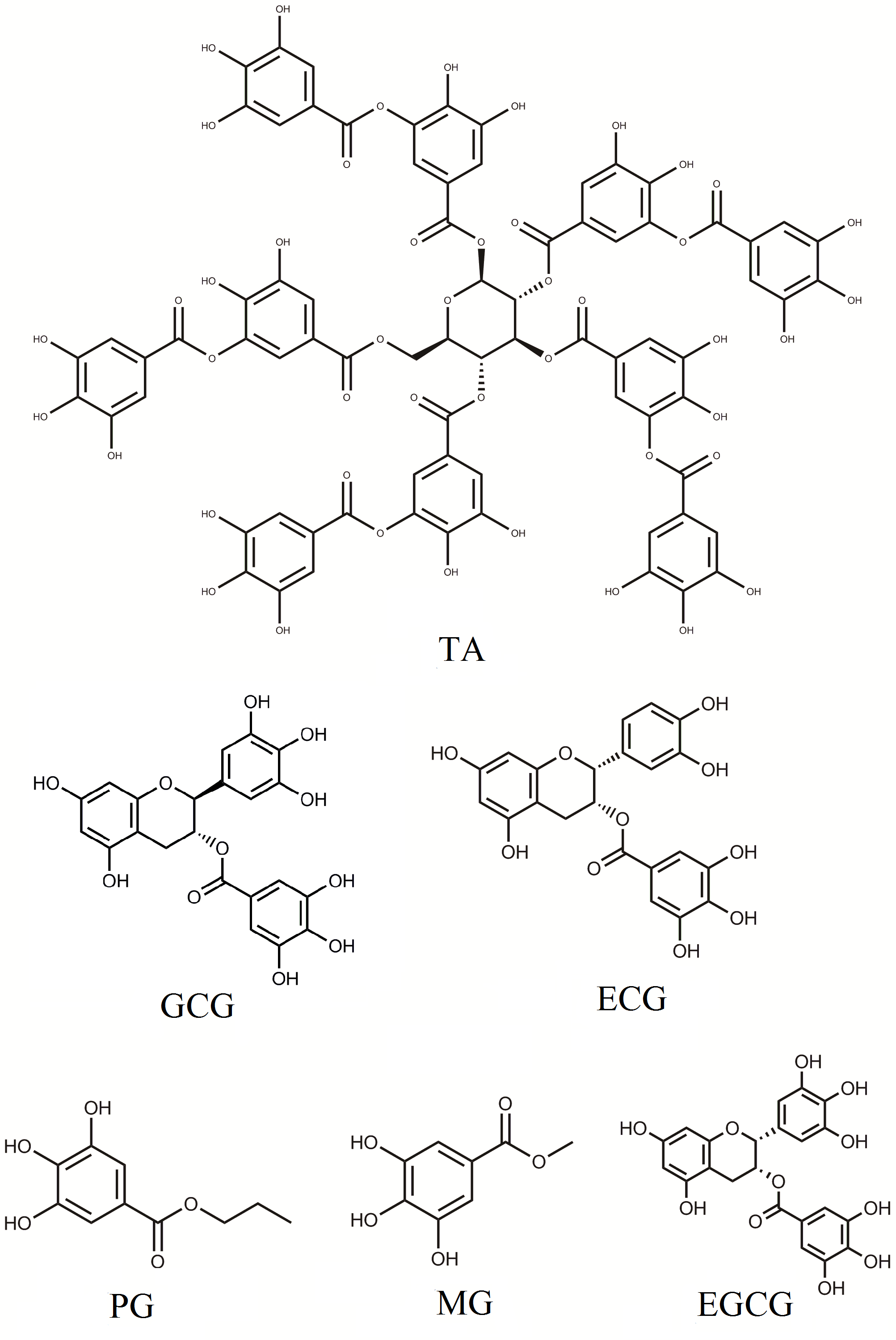


**Figure S1.** Chemical structures of the substrates described in the study. TA: tannic acid; GCG: gallocatechin gallate; ECG: epicatechin gallate; PG: propyl gallate; MG: methyl gallate; EGCG: epigallocatechin gallate.


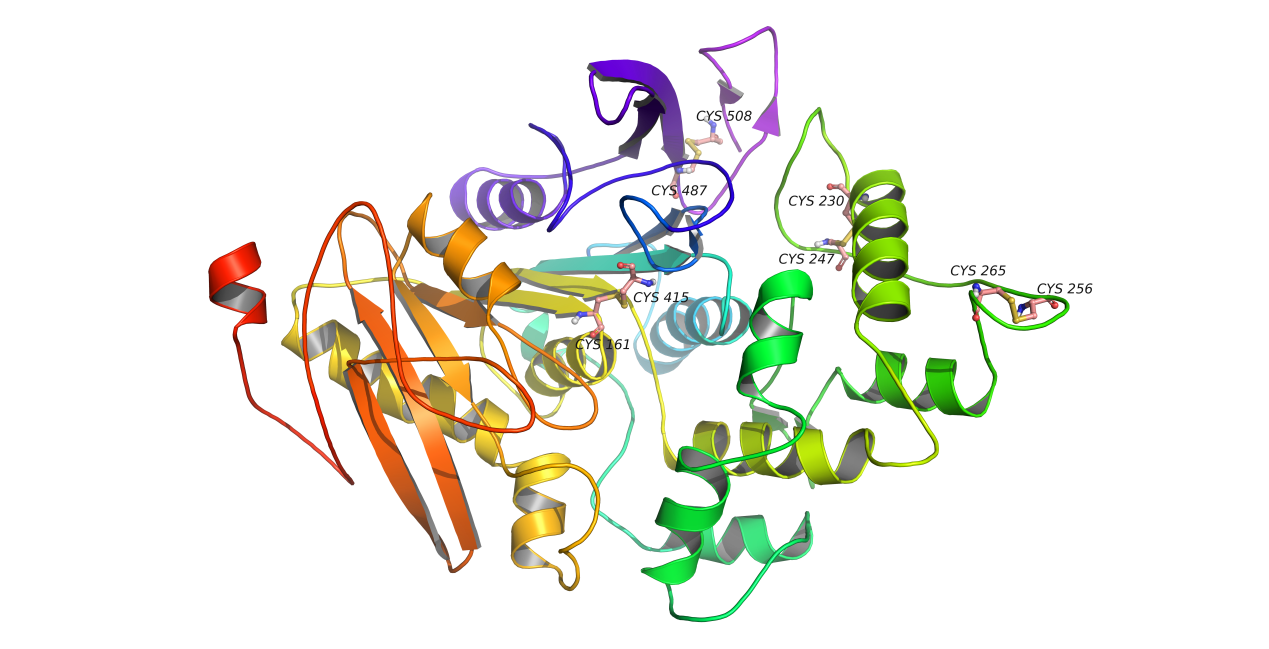


**Figure S2.** 3D structure of TanA based on homology modeling. The predicted formation of four disulfide bonds was shown.


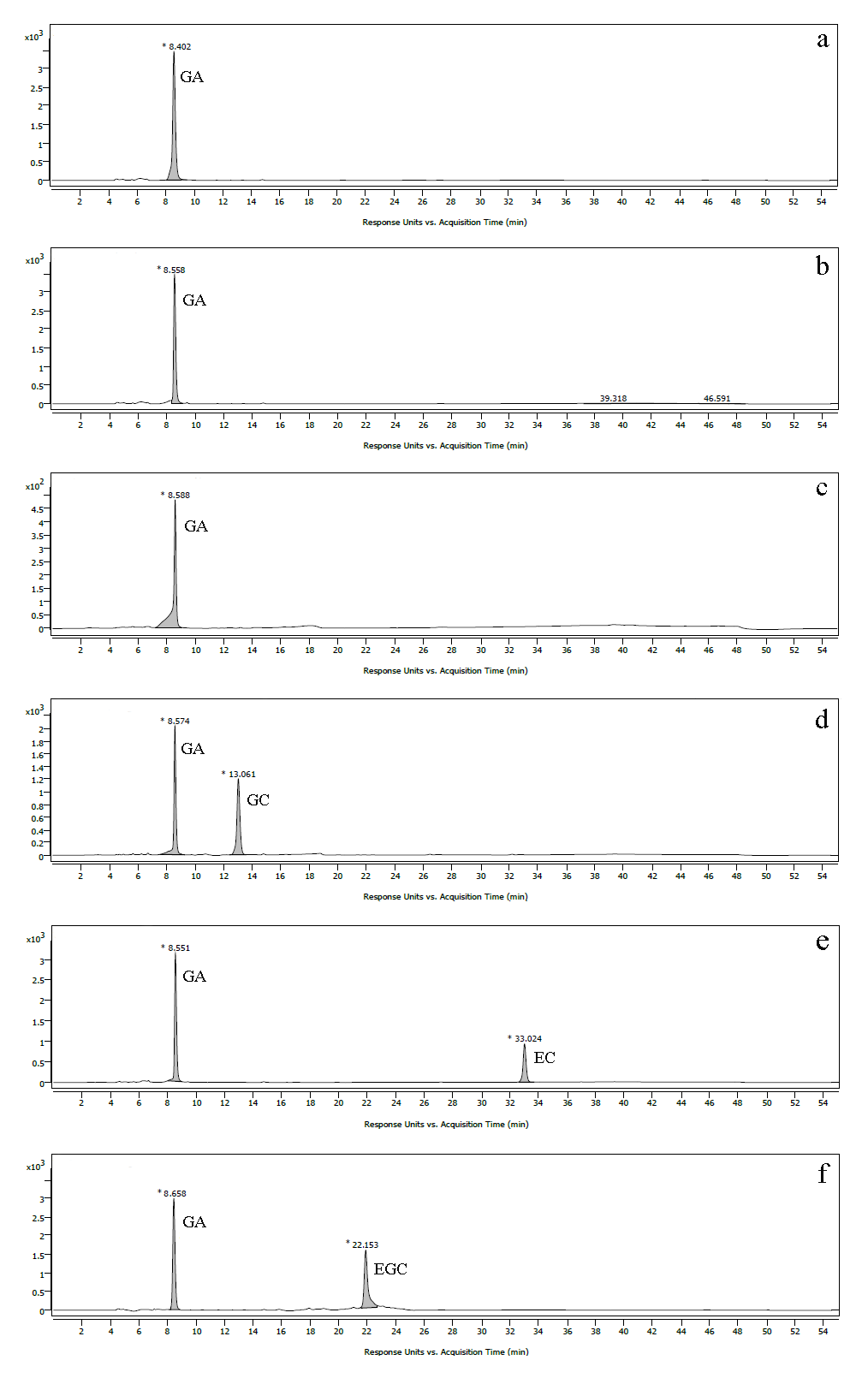


**Figure S3.** Chromatograms of the transformation products after tannase TanA treatment against several gallic acid esters, including MG (a), PG (b), TA (c), GCG (d), ECG (e), and EGCG (f). The degradation products contained GA (gallic acid), GC (gallocatechin), EC (epicatechin), and EGC (epigallocatechin).
